# Halofantrine protects photoreceptors in multiple models of retinal degeneration

**DOI:** 10.21203/rs.3.rs-9511432/v1

**Published:** 2026-05-13

**Authors:** Minji Kim, Anupam K Mondal, Holly Y. Chen, Dheeraj Agrohia, Milton A. English, Phillip Vanlandingham, Florian Regent, Raja Muhammad Naseer Khan, Yi Zeng, Oluwatobi T. Arisa, William D. Figg, Manju Swaroop, Wei Zheng, Wenwei Huang, Rafal Farjo, Hyun Beom Song, Anand Swaroop

**Affiliations:** 1 Neurobiology, Neurodegeneration and Repair Laboratory, National Eye Institute, National Institutes of Health, Bethesda, MD 20892, USA; 2 Department of Biomedical Sciences, Seoul National University College of Medicine, Seoul, Republic of Korea; 3 EyeCRO, Oklahoma City, OK 73105, USA; 4 Clinical Pharmacology Laboratory, Clinical Center, National Institutes of Health, Bethesda, MD, USA; 5 The National Center for Advancing Translational Sciences, National Institutes of Health, Bethesda, MD, 20850, USA

## Abstract

Inherited retinal degenerative diseases (IRDs), including retinitis pigmentosa (RP) and Leber congenital amaurosis (LCA), cause progressive vision impairment due to photoreceptor degeneration. We previously demonstrated the protective effects of reserpine in models of *CEP290*-LCA and in the female *Rhodopsin*-P23H rat model of autosomal dominant RP. Given that reserpine is limited by side effects, we expanded our compound screen using retinal organoids from the *rd16* mouse model of *CEP29*0-LCA to include structurally diverse small molecules that may converge on proteostasis pathways. Follow-up evaluations in *Rhodopsin*-P23H rats, human *CEP290*-LCA retinal organoids, and *rd10* mice (a model of *PDE6B*-RP) identified halofantrine as the most promising molecule, conferring strong protection to both rod and cone photoreceptors and demonstrating therapeutic potential across the divergent IRD models tested. Transcriptome analysis revealed a convergent molecular response to halofantrine across these models, primarily through upregulation of oxidative phosphorylation and ribosomal processes. Notably, halofantrine elicited significant improvements in retinal function, as assessed by electroretinography, despite pharmacokinetically limited and transient retinal exposure after subconjunctival injection in *Rhodopsin*-P23H rats. Our studies highlight the potential of halofantrine as a gene-independent therapy and suggest that it may be developed for ocular delivery via eye drops for the treatment of IRDs.

## Introduction

Neurodegenerative diseases, such as Alzheimer’s disease, Parkinson’s disease, and age-related macular degeneration, affect millions worldwide by causing irreversible deficits in sensory, motor, or cognitive function, and their burden continues to grow as life expectancy increases ^1^. These disorders exhibit vast genetic heterogeneity ranging from rare monogenic variants to largely complex polygenic architectures; nonetheless, all are uniformly characterized by progressive and selective loss of specific neuronal populations as well as pathogenic hallmarks ^2-5^. Therapeutic development remains challenging, in part owing to the limited regenerative capacity of most neurons following injury or degeneration ^6-8^. Therefore, there is a critical need for gene-independent neuroprotective strategies that can preserve neuronal viability across diverse genetic backgrounds.

Loss of vision is the end result of neuronal degeneration in the retina, a specialized visual sensory tissue of the central nervous system ^9^. Inherited retinal degenerative diseases (IRDs) manifest in diverse clinical phenotypes yet share a common hallmark of progressive and selective loss of photoreceptors, the neurons essential for initiating the visual process ^10-12^. IRDs are caused by mutations in over 400 genes ^13-15^; of these, retinitis pigmentosa (RP) and Leber congenital amaurosis (LCA) are among the most prevalent non-syndromic disorders. RP initially affects rod photoreceptors, followed by secondary cone degeneration, and is associated with mutations in over 100 genes with *RHO*, *RPGR,* and *PDE6B* accounting for a significant fraction ^14,16^. LCA is a severe early-onset retinal dystrophy caused by mutations in at least 25 genes, with *CEP290* mutations accounting for approximately 20–30% of cases ^14,17-19^. Despite advances in delineating the genetic basis of IRDs, effective therapeutic options remain limited ^20^.

Recent advancements in gene-based therapy have provided proof of concept for the treatment of several IRDs. Gene replacement therapy, though successful for *RPE65*-associated LCA ^21^, remains inherently gene-specific, and even emerging gene-editing technologies face substantial challenges in scaling across hundreds of distinct genetic etiologies ^22-25^. Other approaches, including neurotrophic factors such as brain-derived neurotrophic factor, ciliary neurotrophic factor, and stem cell-based therapies, face challenges related to delivery, targeting, and durability of effect ^26-29^. Consequently, there is growing interest in small-molecule, gene-agnostic strategies that preserve neuronal viability by targeting shared pathological mechanisms, regardless of the underlying genetic defect ^30-33^.

We previously conducted a high throughput screen of small molecules with the goal of repurposing compounds to protect photoreceptors in IRDs ^34^. We used rod photoreceptors derived from retinal organoids of the *rd16* mouse, a model of LCA caused by a *CEP290* mutation that disrupts cilia development and function and leads to photoreceptor loss ^35^. Our screening of over 6,000 molecules identified reserpine, an antihypertensive drug, as the lead compound that promoted rod photoreceptor survival, which was validated in human *CEP290*-LCA retinal organoids and *in vivo* mouse models ^36^. Follow-up studies in the *Rhodopsin*-P23H transgenic rat model further established reserpine’s potential as a gene-agnostic neuroprotector ^37^. However, reserpine has complex side effects on dopaminergic neurons ^38,39^. Therefore, building upon these findings, we extended the screening to identify superior candidate compounds and evaluated their applicability across distinct RP and LCA disease models. Here, we identify and validate halofantrine as a small molecule for gene-independent treatment of IRDs using a comprehensive pipeline of mouse and rat models, as well as human-derived organoids. Furthermore, we investigate the optimal route of administration, performing a detailed pharmacokinetic evaluation to assess feasibility for clinical translation.

## Results

### Initial screening in *Cep290^rd16/rd16^* mouse retinal organoids

**Fig 1.**
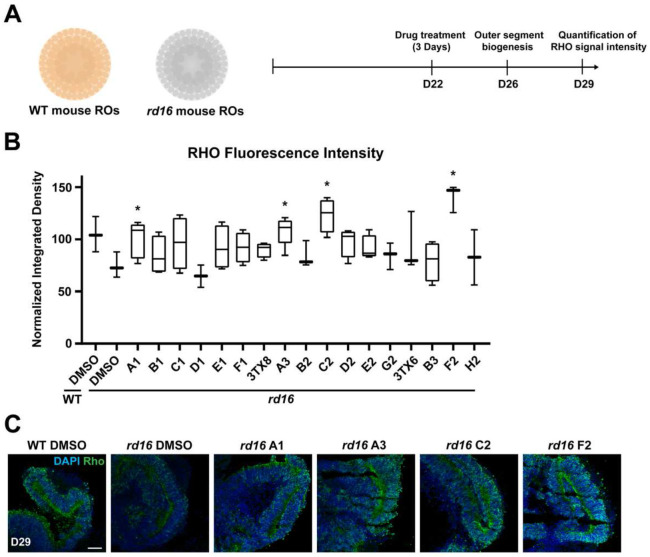
Phenotypic screening and identification of candidate compounds in *rd16* retinal organoids. **(A)** Timeline of compound treatment and organoid harvest. **(B)** The graph shows the quantification of rhodopsin fluorescence intensity in the validation experiment. The bar length represents the distribution of multiple data points, the line across each bar indicates the mean, and the error bars represent the standard error of the mean. **(C)** Immunostaining of rod cell marker rhodopsin (Rho, green) in wild-type (WT) and rd16 organoids treated with positive compounds (A1, A3, C2, F2). Nuclei were stained by 4',6-diamidino-2-phenylindole (DAPI). The scale bar represents 50 μm, *P < 0.05

To identify alternative compounds with improved efficacy and safety beyond reserpine, we selected a panel of candidate small molecules based on our prior mechanistic insights. We evaluated 17 small molecules (Table S1), including reserpine (A1), its structural analogs, and compounds targeting lysosome-associated proteostasis pathways using *rd16* mouse retinal organoids ^40^ to identify candidates that improve photoreceptor development or survival. Following our previous screening scheme (Fig. 1A) ^36^, the molecules were treated from day 22 to 25, and the organoids were harvested on day 29 for immunostaining using anti-rhodopsin antibody. Rhodopsin, a key protein essential for phototransduction in rod photoreceptors, was used to assess photoreceptor structure and survival. Among the 17 small molecules tested, A3, C2, and F2 were identified as the most promising candidates based on increased rhodopsin fluorescence intensity, demonstrating significantly higher efficacy than reserpine (A1) (Fig. 1B,C). In contrast, compounds that did not produce a comparable increase in rhodopsin fluorescence intensity were considered less effective than reserpine and were not chosen for further study.

### Assessment of candidate molecules in human *CEP290*-LCA retinal organoids

**Fig 2.**
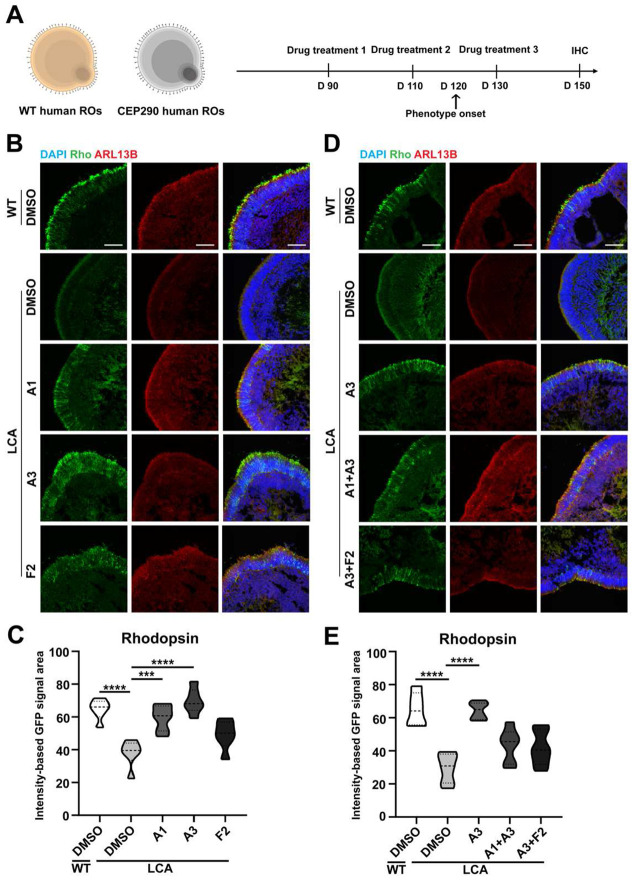
Validation of selected candidates in LCA10 patient-derived retinal organoids. **(A)** Timeline for compound treatments and harvest of patient organoids. **(B,D)** Immunostaining of rhodopsin (Rho, green). Nuclei were stained with 4',6-diamidino-2-phenylindole (DAPI). Images are representative of at least three independent groups, each containing at least four organoids. The scale bar represents 100 μm. **(C,E)** Violin plots show the quantification of fluorescence intensity from rhodopsin staining in the validation dataset. The shape of each plot represents the distribution of individual data points. The scale bar represents 50 μm, ***P < 0.001, ****P < 0.0001.

Based on the outcome of the initial screening, we validated A3, C2, and F2, along with reserpine (A1) in iPSC-derived retinal organoids from an LCA10 patient family, including an unaffected mother (control) and an affected compound heterozygous child ^36,41^. Given that photoreceptor development and outer segment biogenesis in retinal organoids initiate around day 120 (D120), and abnormal phenotypes in patient-derived organoids become apparent at this stage ^42^, we initiated treatment at D90 to intervene before these developmental milestones (Fig. 2A). Based on the concentration used for screening in *rd16* organoids (Fig. 1), we treated the human organoids with A1 at 30 μM and A3, C2 and F2 at 10 μM. During the treatment period, media were replenished every 2 to 3 days to maintain optimal culture conditions. Both A1 and A3 significantly increased rhodopsin fluorescence intensity in patient-derived retinal organoids, indicating enhanced rod photoreceptor survival compared with DMSO-treated controls (Fig. 2B,C). F2 exhibited a trend toward increased rhodopsin expression that did not reach statistical significance, whereas C2 showed cellular toxicity and was excluded from further assays (Fig. S1). Given the established role of CEP290 in ciliogenesis, we evaluated ARL13B to assess the restoration of ciliary morphology ^41^; partial improvements were observed in both A1- and A3-treated groups. Subsequently, we evaluated combinations of A3 (the candidate with the highest efficacy) with other candidates at lower concentrations (A3+A1 or A3+F2). However, these combinations did not confer additional augmentation of rhodopsin immunoreactivity compared to A3 alone (Fig. 2D,E). Importantly, no adverse effects were observed on cone photoreceptors, synaptic structures, or bipolar cells following treatment with any of the drugs or their combinations (Fig. S2 and Fig. S3).

### Functional and histological evaluation of candidate molecules in P23H rats

**Fig 3.**
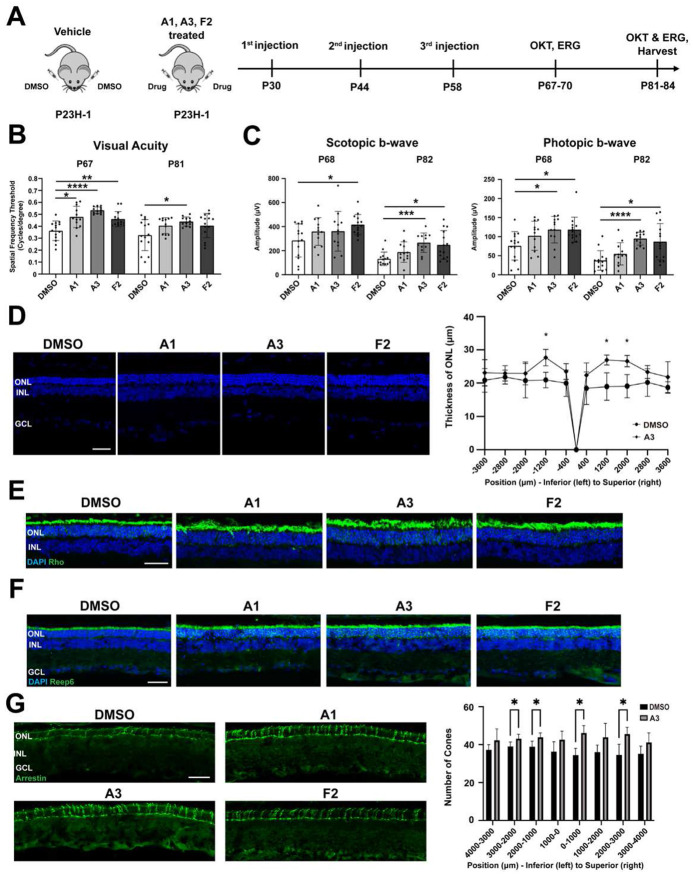
Functional and structural preservation by potent candidates in P23H rats. **(A)** Timeline of in vivo intravitreal injections for functional and structural studies. **(B)** Visual acuity measured by OKT in DMSO- and compound-treated P23H rats was assessed in both eyes (7 DMSO-treated and 6 or 7 compound-treated rats). **(C)** Scotopic and photopic b-wave ERG responses at P68 and P82 in DMSO- and compound-treated P23H rats. Data were expressed as mean ± SEM, using Brown-Forsythe and Welch ANOVA tests. **(D)** ONL thickness evaluated by DAPI-stained retinal sections at P84. The representative images were taken from the retina where the most significant changes were observed. Images are representative of six or seven female rats per group. **(E,F)** Rod photoreceptors were evaluated by immunostaining for REEP6 and rhodopsin (Rho, green) at P84. **(G)** Cone photoreceptors were evaluated by immunostaining of cone arrestin at P84. Numbers of cone photoreceptors were counted every 1000 μm throughout the retina. Data were expressed as mean ± SEM, using Mann-Whitney U test. The scale bar represents 40 μm. ONL: outer nuclear layer, INL: inner nuclear layer, GCL: ganglion cell layer, ERG: electroretinogram, OKT: Optokinetic tracking test, *P < 0.05, **P < 0.01, ***P < 0.001, ****P < 0.0001.

To further evaluate the top compounds across IRD models, we selected reserpine (A1), A3 and F2 for assays of retinal function and visual performance using the well-characterized *Rhodopsin*-P23H rat model of autosomal dominant RP ^43-45^. We followed the previously established protocol ^37^ to examine therapeutic efficacy in female P23H rats but extended the treatment window by adding a third intravitreal injection at postnatal day 58. Following intravitreal injections, optokinetic tracking (OKT) was performed at P67 and P81, and electroretinography (ERG) was performed at P68 and P82 (Fig. 3A). In OKT measurements at P67, treatments with A1, A3 and F2 revealed significantly improved visual performance, with up to 1.5-fold increase compared to the control group (Fig. 3B). However, the effect was sustained at P81 only in the A3-treated group. ERG assays revealed that, at P68, F2 treatment significantly increased the scotopic b-wave compared with the DMSO control, whereas both A3 and F2 enhanced photopic b-wave responses (Fig. 3C). As retinal degeneration progressed to P82, the neuroprotective effects became more pronounced; both A3 and F2 significantly preserved scotopic and photopic b-wave amplitudes, with A3 showing the most robust improvement (Fig. 3C). However, there were no significant changes in scotopic or photopic a-wave amplitudes at P68 or P82 (Fig. S4A).

We then examined retinal histology of *Rhodopsin*-P23H rats after treatment with A1, A3 and F2 and assessed rod preservation at P84 by measuring the thickness of the outer nuclear layer (ONL) ^46^. All three molecules significantly preserved ONL thickness, but A3 showed the most pronounced effect (Fig. 3D and Fig. S4B). To further evaluate rod photoreceptor integrity, we performed immunostaining for REEP6 and rhodopsin, two established rod markers ^47^. Consistent with rod preservation, A3 exhibited the highest REEP6 and rhodopsin immunostaining (Fig. 3E,F). Notably, similar results were observed with anti-cone arrestin antibody ^48^ after treatment with A1, A3 and F2, with A3 showing the best cone preservation (Fig. 3G and Fig. S4C).

A3 was identified as halofantrine, an FDA-approved antimalarial agent and was selected as the lead candidate for further *in vivo* evaluation.

### Halofantrine protects photoreceptors in the *rd10* mouse retina

**Fig 4.**
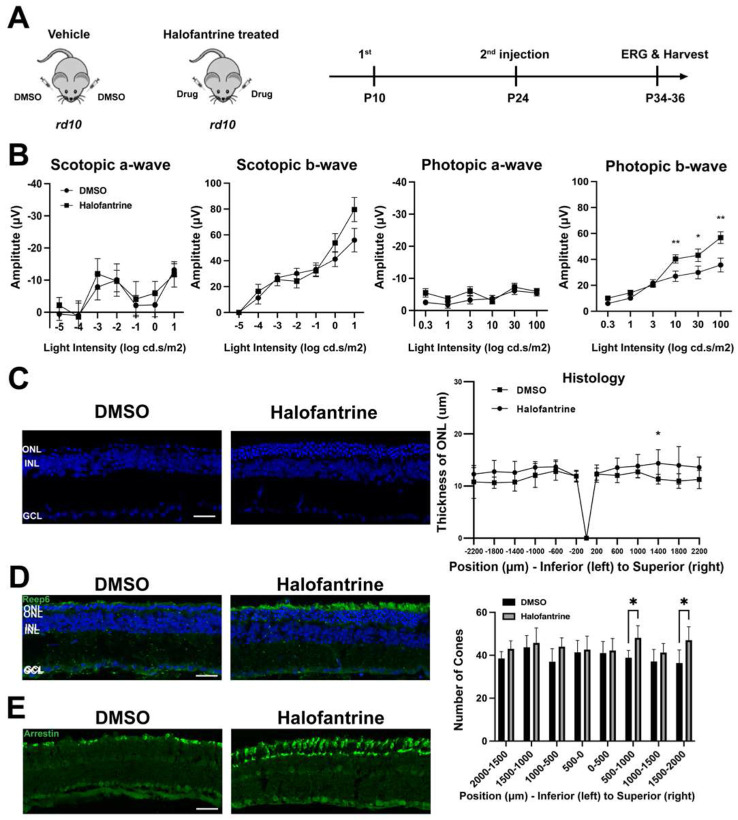
Therapeutic efficacy of halofantrine in the *rd10* mice. **(A)** Timeline of in vivo intravitreal injections for quantification of outer nuclear layer thickness (ONL), retinal morphology, and immunohistochemistry studies. **(B)** Scotopic and photopic electroretinogram responses in DMSO-treated and halofantrine-treated *rd10* mice. **(C)** Outer nuclear layer thickness evaluated by DAPI-stained retinal section at P36. The representative images were taken from superior retina, 1800 μm away from the optic nerve. Data were expressed as mean ± SEM, and the Mann-Whitney U test was used to compare DMSO- and Halofantrine-treated groups. Images are representative of ten mice. **(D)** Rod photoreceptors were evaluated by immunostaining for REEP6 (green) at P36. **(E)** Cone photoreceptors were evaluated by immunostaining of cone arrestin at P36. Numbers of cone photoreceptors were counted every 500 μm throughout the retina. Data were expressed as mean ± SEM, and the Mann-Whitney U test was used to compare DMSO- and halofantrine-treated groups. Representative images were taken from retinal regions where the most significant changes were observed. The scale bar represents 20 μm. *P < 0.05

We next evaluated the therapeutic potential of halofantrine in the *rd10* mouse, which exhibits a well-characterized retinal degeneration phenotype caused by a mutation in the *Pde6b* gene ^49^. Given that photoreceptor degeneration begins around P16 in the *rd10* retina ^50^, halofantrine (20 μM) was administered via intravitreal injection at P10 and P24, followed by ERG evaluation at P34, and retinas were collected at P36 (Fig. 4A). ERG measurements showed that halofantrine treatment significantly improved photopic b-wave responses compared to the control group (Fig. 4B). While scotopic b-wave responses showed a trend toward augmentation, the difference was not statistically significant, and no improvements were observed in either scotopic or photopic a-waves. Additionally, halofantrine preserved photoreceptors, as evidenced by a significant increase in ONL thickness (Fig. 4C and Fig. S5). We also detected increased REEP6 expression at P36, consistent with rod preservation after halofantrine treatment compared to *rd10* DMSO-treated mice (Fig. 4D). Furthermore, cone arrestin-positive cells were significantly enhanced in halofantrine-treated mouse retinas (Fig. 4E). Wild-type control mouse retina was included for comparison in Fig. S6.

### Shared transcriptomic responses to halofantrine across retinal disease models

**Fig 5.**
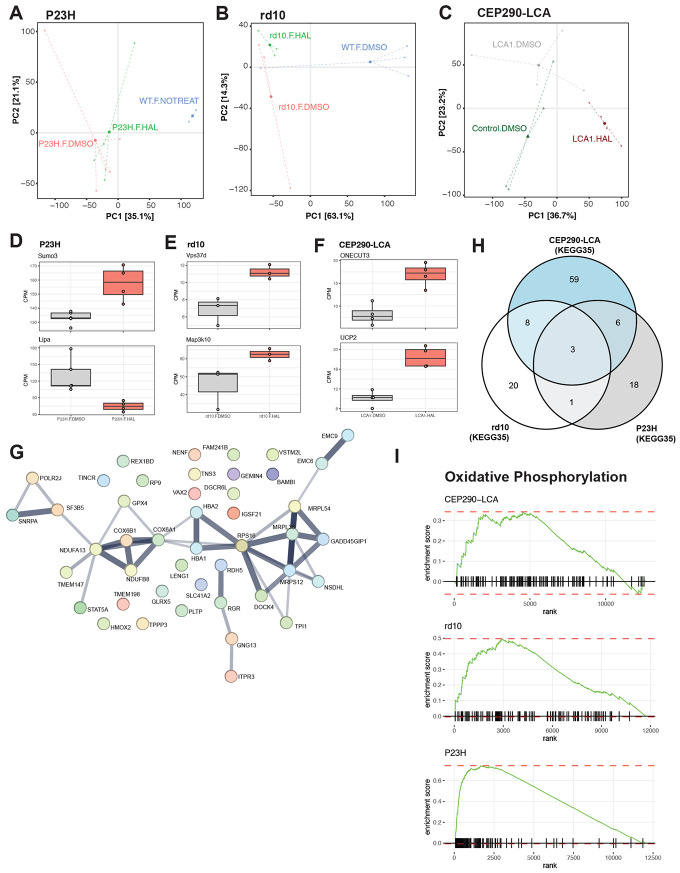
Halofantrine induces comparable transcriptomic changes in distinct retinal disease models. **(A,B,C)** Principal component analysis (PCA) plot summarizing tanscriptome remodulation in P23H female rat retinas, rd10 female mice and CEP290-LCA retinal organoids. **(D)** Expression changes in Sumo3 and Lipa genes after halofantrine treatment in P23H rat retinas. **(E)** Vps37d and Map3k10 genes significantly respond to Halofantrine in female rd10 retinas. **(F)** CEP290-LCA retinal organoids show marked difference in ONECUT3 and UCP2 gene expression. **(G)** Network showing predicted protein-protein interaction among gene products of shared halofantrine responsive genes. **(H)** Comparison of KEGG pathways mapped to DEGs reveal overlapping functional perturbation in distinct retinal disease models in response to halofantrine. **(I)** Enrichment plot summarizing overexpression of OXPHOS related genes after halofantrine treatment.

To gain molecular insights into photoreceptor neuroprotection by halofantrine, we generated global transcriptome profiles of the three retinal disease models (*Rhodopsin*-P23H rats, *rd10* mice, and *CEP290*-LCA human retinal organoids) with and without drug treatment. Halofantrine treatment markedly altered gene expression patterns in all disease models tested in our study (Fig. 5). In mutant P23H rats, PCA analysis revealed drug-injected female retinas to be distinct from untreated mutants and closer to untreated WT females (Fig. 5A). Retinal transcriptomes of *rd10* female mice shifted upon halofantrine treatment highlighting a consistent *in vivo* response (Fig. 5B). We also noted similar halofantrine sensitivity in human *CEP290*-LCA retinal organoids (Fig. 5C). The most significant halofantrine responsive genes in *Rhodopsin*-P23H retinas were *Sumo3* and *Lipa*; in *rd10* retinas they were *Vps37s* and *Map3k10*, and in human *CEP290*-LCA retinal organoids they were *ONECUT3* and *UCP2* (Fig. 5D,E,F).

Given that all three models represent different mammalian species and likely distinct disease mechanisms, we decided to identify specific and shared functional changes driven by halofantrine using a two-pronged approach – first, by comparing differential gene orthologs across the disease models, and the second, by mapping differential genes to the KEGG database before examining overlaps at the pathway level. Gene level analysis revealed 46 shared differential genes in at least two disease models after halofantrine treatment (Fig. S7A). Interestingly, the *ITPR3* gene showed a significant response to halofantrine in all three disease models (Fig. S7B). Mapping to the STRING protein-protein interaction database revealed functional associations within a subset of the shared differential genes (Fig. 5G). Pathway analysis highlighted a set of 18 pathways perturbed by halofantrine in two or more disease models (Fig. 5H, Fig. S7C). Oxidative phosphorylation (OXPHOS), ribosome and circadian entrainment were noted in all models. Enrichment analysis confirmed halofantrine-linked upregulation in OXPHOS and ribosomal processes across the three retinal disease models (Fig. 5I, Fig. S7D). We also detected limited targeting of proteasomal processes by gene expression changes (Fig. S7E). Overall, halofantrine-associated gene expression responses exhibited partial similarity across distinct retinal disease models.

### Investigations of optimal halofantrine delivery route in rats

**Fig 6.**
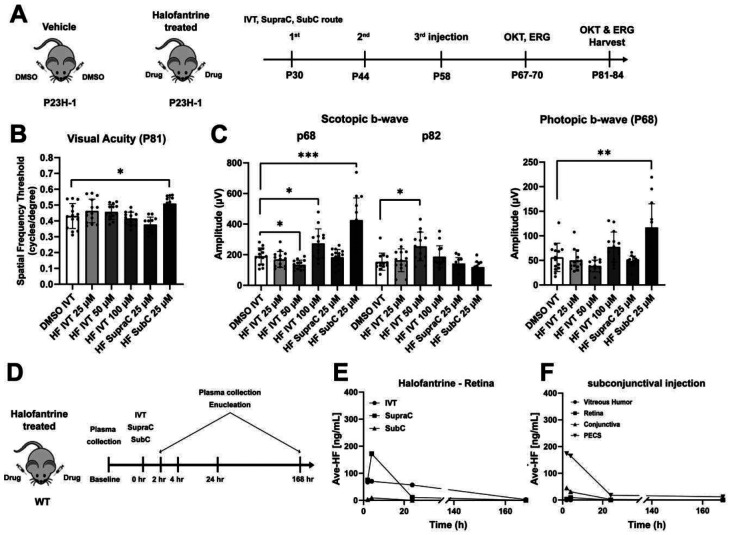
Impact of delivery routes on halofantrine efficacy in rats **(A)** Timeline of in vivo intravitreal, suprachoroidal, and subconjunctival injections for functional studies in P23H rats. **(B)** Visual acuity measured by OKT in both eyes of P23H rats treated with DMSO (n=7) or halofantrine at different doses and delivery routes (n=7). Data were expressed as mean ± SEM, and the Brown-Forsythe and Welch ANOVA tests were used to compare the groups. **(C)** Scotopic and photopic ERG responses at P68 and P82 in DMSO- and halofantrine-treated P23H rats with three different delivery routes and doses. **(D)** Timeline of in vivo administration of halofantrine via different ocular routes for pharmacokinetic analysis of ocular tissue distribution and retention in WT rats. **(E)** Retina pharmacokinetics and tissue drug distribution following three different delivery routes in WT rats. **(F)** Halofantrine distribution across ocular tissues following subconjunctival injection. Animals were euthanized at the specified time points. HF: halofantrine, PECS: RPE/choroid/sclera, IVT: Intravitreal injection, SupraC: suprachoroidal injection SubC: subconjunctival injection *P < 0.05, **P < 0.01, ***P < 0.001

Building on the demonstrated efficacy of halofantrine in improving retinal function and photoreceptor survival, we investigated different routes of administration by evaluating functional outcomes in female P23H rats and pharmacokinetics in wild-type rats. Following the experimental timeline (shown in Fig. 3A), we evaluated both the original halofantrine concentration and higher doses, administering these through intravitreal, subconjunctival as well as suprachoroidal routes in female P23H rats (Fig. 6A). To further evaluate visual performance, OKT measurements of the spatial frequency threshold were performed and revealed a marked improvement in visual acuity at P81 following a 25 μM subconjunctival injection (Fig. 6B). Notably, an intravitreal dose of 50 μM initially reduced the scotopic b-wave amplitude at P68, with significant recovery by P82, consistent with a gradual decrease in intraocular drug concentration (Fig. 6C). In contrast, a higher intravitreal dose of 100 μM produced greater functional improvement in the scotopic b-wave at P68 without an initial suppression. These findings suggest a non-linear, dose- and time-dependent biphasic response. We also observed that a 25 μM subconjunctival injection further enhanced both scotopic b-wave and photopic b-wave response at P68. However, no significant changes were observed in scotopic or photopic a-wave amplitudes at P68 or P82, nor in photopic b-wave responses at P82 or visual acuity at P67 (Fig. S8A, B).

To evaluate the pharmacokinetics of halofantrine in the eye, we compared its distribution and retention in ocular tissues following the same concentration but different routes of administration and timepoints in wild-type rats (Fig. 6D). Halofantrine concentrations in plasma were below the lower limit of quantification (1 ng/mL) across all groups, indicating minimal to no systemic exposure (Table S2). In the retina, suprachoroidal administration produced the highest peak concentration at 4 hours and was eliminated more rapidly, whereas intravitreal injection exhibited slower retinal clearance and higher concentrations at 24 hours. Subconjunctival injection yielded low to undetectable concentrations in the retina at all time points (Fig. 6E). In the vitreous, suprachoroidal injection reached the highest peak concentration, followed by intravitreal injection, while subconjunctival delivery remained undetectable (Fig S9A). Conversely, subconjunctival administration resulted in the highest drug levels in the retinal pigment epithelium (RPE) /choroid/sclera (PECS) (Fig. 6F), whereas intravitreal and suprachoroidal injections yielded largely minimal concentrations in these tissues (Fig. S9B,C). We note that halofantrine produced significant functional improvements despite substantially reduced retinal concentrations at 24 hours across all delivery routes, highlighting its therapeutic potential even at relatively low ocular exposures.

## Discussion

Retinal degenerative diseases place a substantial burden on patients by impairing quality of life, independence, and productivity ^51^. Although significant progress has been made in identifying IRD-associated genes and elucidating pathogenic mechanisms, the extensive genetic heterogeneity continues to limit the broad application of gene-targeted therapies ^52^. Previously, we identified reserpine as a lead compound that promotes photoreceptor survival in CEP290-related ciliopathy, potentially through modulation of proteostasis and autophagy pathways ^36^. Reserpine’s gene-independent therapeutic potential was demonstrated in *Rhodopsin*-P23H transgenic rat model of adRP ^37^. However, reserpine’s translational potential may be limited because of neuropsychiatric effects and clinical tolerability. Here, we identify halofantrine as a superior compound with gene-independent efficacy while offering improved safety and broader therapeutic applicability.

Halofantrine treatment revealed significantly higher rhodopsin immunostaining in retinal organoids derived from a *CEP290*-LCA patient as well as those from the corresponding *rd16* mouse model. Halofantrine alone outperformed the combination treatments with reserpine or F2. In the P23H transgenic rat model *in vivo*, halofantrine treatment led to significant improvements in both scotopic and photopic ERG responses, alongside enhanced visual performance measured by optokinetic tracking (OKT) at P67, with sustained effects observed at P81 (even three weeks after the treatment). Broad photoreceptor protection was evident by significant preservation of ONL thickness and increased expression of rhodopsin and REEP6, as well as enhanced cone arrestin labeling. *In vivo* validation in the *rd10* mouse model further confirmed the gene-independent therapeutic efficacy of halofantrine, demonstrating improved retinal function by ERG, preservation of ONL thickness, and survival of both rod and cone photoreceptors following intravitreal administration.

Transcriptome analyses of disease models treated with halofantrine showed consistent trends, suggesting that the disease gene and mutation independent neuroprotection offered by halofantrine is orchestrated in part by similar molecular perturbations. Both gene and pathway level investigations uncovered common elements in halofantrine activity. One gene (*ITPR3*) responded in all tested disease models, and several others were present in more than one pathology, pointing to consistent molecular partners mediating halofantrine activity. Further support for this hypothesis comes from the overlap in pathways impacted by halofantrine. We therefore suggest that halofantrine is a promising gene-agnostic therapeutic candidate for genetically diverse IRDs.

Notably, halofantrine-induced upregulation of OXPHOS was consistently identified across all our experimental models (Figure 5I). Rod photoreceptors, the primary cells affected in RP, rely both on aerobic glycolysis as well as mitochondrial OXPHOS because of high energy demands and are selectively vulnerable to decoupling of the electron transport chain, ^53^. We had previously provided evidence of early and progressive mitochondrial stress, along with structural and functional abnormalities, as initial drivers of photoreceptor cell death in *rd1* mouse model of RP ^54^. Furthermore, mitochondrial and metabolic dysfunctions have been implicated in age-related macular degeneration ^55,56^ and glaucoma ^57,58^. Halofantrine may therefore exert neuroprotective effects by modulating shared downstream mechanisms, supporting its potential as a gene-independent therapeutic candidate for IRDs and other neurodegenerative diseases.

Though FDA-approved, halofantrine is no longer available in the US because of systemic safety concerns regarding cardiotoxicity, specifically QTc interval prolongation, at the recommended therapeutic doses ^59,60^. Nonetheless, we here demonstrate that localized ocular delivery confers robust neuroprotective effects across multiple models of retinal degeneration. Notably, even at doses showing high efficacy, none of the localized delivery routes result in detectable plasma concentrations (Table S2). By minimizing systemic absorption through the periocular route, we have been able to preserve therapeutic efficacy while effectively bypassing known cardiotoxicity. These results strongly argue in favor of repurposing halofantrine via localized delivery for the treatment of retinal diseases and provide a rationale for further investigation into its ocular therapeutic applications.

Invasive administration routes, such as intravitreal and suprachoroidal injections, enable direct delivery to the retina bypassing physical barriers, thereby achieving high local concentrations. Our pharmacokinetic results demonstrate that halofantrine is effective at very low concentrations even with short-term retinal presence following the intravitreal injection. Interestingly, subconjunctival administration elicits the most robust functional improvements in the P23H rat model, surpassing even intravitreal delivery, as evidenced by significantly enhanced ERG responses and visual acuity in OKT assessments. This superior efficacy via the subconjunctival route may be attributed to several factors. First, the subconjunctival space can act as a local depot, allowing the drug to gradually diffuse across the sclera into the posterior segment ^61^. Although peak intraocular concentrations remain lower than those achieved with intravitreal delivery, trans-scleral diffusion may provide more persistent, steady-state exposure to the neural retina, which appears more effective for halofantrine’s therapeutic action. Second, subconjunctivally administered drugs may directly reach and modulate the function of RPE ^62^, which is critical for photoreceptor survival through nutrient transport and metabolic support. Therapeutic effects may therefore be attributed to indirect RPE-mediated modulation of photoreceptor protection, even when direct drug levels within the neural retina are minimal.

Our studies suggest that halofantrine can exert neuroprotective effects via less invasive routes, supporting its potential as a safe, locally delivered ocular therapeutic and informing future development of topical formulations such as topical eye drops. Nonetheless, poor aqueous solubility of halofantrine remains a challenge ^63^. In addition, given the known cardiotoxicity associated with high systemic exposure ^59,60^, localized application and precise dose optimization will be essential to maximize the therapeutic window. Further investigations to refine delivery strategies and define safe and effective exposure ranges are ongoing for clinical translation of this promising neuroprotective drug candidate.

## Material and Methods

### Animals

Hemizygous P23H-1 rats were generated and previously characterized through in-house breeding at EyeCRO by crossing transgenic P23H line 1 rats with wild-type Long Evans rats obtained from Charles River, in order to model the clinical progression of adRP ^37^. Animals were housed in ventilated cages in groups of 2-3 under controlled 12-hour light/dark cycle, with free access to food and water. All experimental procedures were conducted in accordance with the ARVO Statement for the Use of Animals in Ophthalmic and Vision Research and were approved by the Institutional Animal Care and Use Committee (IACUC; Protocol No. 2021-10-16-001).

B6.CXB1-*Pde6b^rd10^*/J (Strain #: 004297; RRID: IMSR_JAX:004279), including both female and male, were obtained from the Jackson Laboratory and intercrossed to generate the pups. All mouse experiments were approved by the Animal Care and Use committee of the National Eye Institutes (animal study protocol NEI-650) and adhered to ARVO Statement for the Use of Animals in Ophthalmic and Vision Research. Mice were maintained under controlled environment conditions (temperature: 22 °C ± 2 °C, humidity: 30-70%) with a 12-hour light/dark cycle and had ad libitum access to food and water. Bedding, food, and water were replaced on a weekly basis.

### ERG recordings

Rats were dark-adapted for a minimum of 12 hours prior to recordings. Under dim red illumination (<50 lux), anesthesia was induced using ketamine (85 mg/kg) and xylazine (4 mg/kg). ERG signals were acquired with the Diagnosys Celeris platform. Scotopic responses were elicited using a flash intensity of 40 cd·s/m^2^ applied to dilated eyes. The a-wave amplitude was defined as the difference between baseline and the trough, while the b-wave amplitude was measured from the a-wave trough to the peak of the b-wave. For photopic recordings, animals were light-adapted for 7 min prior to stimulation with 15 repeated flashes at 10 cd·s/m^2^. The resulting traces were averaged, and b-wave amplitudes under photopic conditions were measured from the a-wave minimum to the b-wave maximum.

For mouse ERG recordings, animals were dark-adapted overnight and anesthetized by intraperitoneal injection of ketamine (100 mg/kg) and xylazine (10 mg/kg). A reference electrode was inserted into the oral cavity, while corneal hydration was maintained using a 2.5% (wt/vol) hypromellose ophthalmic solution (Gonak; Akorn). ERG signals were captured with the Espion E2 Visual Electrophysiology System (Diagnosys) utilizing gold loop electrodes placed on the corneal surface. Scotopic responses were recorded across a flash intensity range of 0.0001 to 10 cd·s/m^2^, with inter-stimulus intervals adjusted between 5 and 60 seconds according to the intensity. Photopic measurements were subsequently performed at intensities from 0.3 to 100 cd·s/m^2^ under rod-saturating background light following a 2-minute light adaptation period. Amplitudes of a- and b-waves were defined as described above. Animals displaying any ocular or lens damage post-injection were excluded from the analysis.

### OKT

Animals were placed on a central platform enclosed by four LCD monitors within a light-controlled chamber. Optokinetic responses were evaluated by a masked observer using a digital camera mounted above the setup. Rotating vertical sine-wave gratings at 12 degrees/s were presented, generating the perception of a virtual cylinder surrounding the animal. A tracking response was characterized by steady and continuous head movements aligned with the direction of the rotating grating stimulus. Spatial frequency thresholds were determined across a range of 0.064 to 0.514 cycles/degree, with the OptoMotry system automatically adjusted stimulus settings according to observer feedback. Contrast sensitivity was evaluated at 0.064 cycles/degree and expressed as the inverse of Michelson contrast, calculated using the formula (maximum − minimum)/(maximum + minimum). Subjects that failed to exhibit tracking behavior were assigned a threshold value of 0.

### Intravitreal injection

P23H-1 rats underwent anesthesia via intraperitoneal injection of ketamine (85 mg/kg) and xylazine (4 mg/kg) cocktail, followed by pupillary dilation using standard ophthalmic procedures. Intravitreal injections were carried out under a stereomicroscope to allow precise positioning of the needle. A volume of 5 μL (20-100 μM) was introduced into the vitreous cavity via the pars plana using a 33-gauge needle attached to a Hamilton syringe. Bilateral injections were performed in all animals.

For intravitreal injections in mice at P10 and P24, pups received intraperitoneal anesthesia with ketamine (50 mg/kg body weight) and xylazine (5 mg/kg body weight). Either DMSO or Halofantrine (1 μL, 20 μM) was delivered into the vitreous using a Hamilton syringe fitted with a 34-gauge needle. Mice showing evidence of ocular or lens injury following injection were excluded from further analysis. All procedures were conducted at the NEI, National Institutes of Health, in accordance with approved protocols from the NEI Animal Care and Use Committee (NEI-ASP650).

### Suprachoroidal injection

Animals were anesthetized with ketamine (85 mg/kg) and xylazine (4 mg/kg), and eyes were dilated following topical application of proparacaine 5 min prior to injection. After anesthesia, animals were positioned on a temperature-controlled heating pad, and the temporal scleral region was visualized under magnification. A 31-gauge insulin syringe (8 mm) was used to create a scleral entry point approximately 1.0-1.5mm below the corneal limbus, taking care to avoid damage to the RPE/Choroid, retina, or vitreous. Injections were then performed using a 10 μL Hamilton syringe equipped with a 33-gauge 45° beveled needle, inserted through the scleral opening with the bevel oriented toward the cornea. At each timepoint, all animals received bilateral injections, with each eye receiving two separate 3 μL doses (a total of 6 μL per eye) at a concentration of 25 μM.

### Subconjunctival injection

Animals were anesthetized using ketamine (85 mg/kg) and xylazine (4 mg/kg), followed by pupillary dilation. Subconjunctival injections were performed using an 8 mm 31G insulin syringe. The needle was inserted such that the conjunctival tissue stretched over the tip upon entry and then advanced carefully until the bevel was fully covered by the conjunctiva prior to injection. At each timepoint, all animals received bilateral injections of 20 μL (25 μM) per eye.

### Pharmacokinetics

Pharmacokinetic analyses were conducted in adult Brown Norway rats (n = 45). Animals were divided into three groups and received bilateral administration of halofantrine (25 μM, 5 μL per eye) through intravitreal, suprachoroidal, or subconjunctival routes. Plasma samples were collected at baseline and at designated terminal time points. Ocular tissues, including vitreous humor, retina, retinal pigment epithelium/choroid/sclera (PECS), and conjunctiva, were harvested at 2, 4, 24, and 168 hours post-administration (n = 3 rats per group per time point). Drug concentrations were quantified using validated LC–MS/MS methods with halofantrine-d9 as an internal standard. The lower limits of quantification were 1 ng/mL for plasma and 0.02 ng/mg for ocular tissues, except for conjunctiva (0.005 ng/mg). Measurements below these thresholds were reported as below the limit of quantitation (BQL). Mean concentration values at each time point were used to generate time-concentration profiles, and all analysis and graphical representations were performed using GraphPad Prism.

### Human pluripotent cell lines

Human induced pluripotent stem cell (iPSC) lines were generated from fibroblasts obtained from a familial control individual and an LCA10 patient (designated LCA-1), derived from skin biopsy samples. Reprogramming was performed using a non-integrating Sendai virus system at the iPSC Core Facility of the National Heart, Lung, and Blood Institute, following established protocol ^41,64^.

### Maintenance of iPSC

Human iPSC lines were cultured in mTeSR^™^1 Basal Medium (STEMCELL Technologies) on plates coated with hESC-qualified Matrigel (Corning). Following daily media replacement, cells were kept under controlled conditions (37 °C, 5% O_2_, 5% CO_2_). Once a confluency of 60–80% was achieved, cells were passaged using an EDTA-based dissociation method.

### Differentiation of human retinal organoids

Retinal organoids were generated from human iPSC using a modified differentiation protocol as previously described ^65^. Briefly, small cell aggregates obtained from one well of a six-well plate were resuspended in mTeSR^™^1 basal medium supplemented with 10 μM Y-27632 (Tocris) and transferred onto a 10cm hESC-qualified Matrigel-coated dish to initiate differentiation. From day 0 to day 3 (D0-D3), cultures were maintained in neural induction medium (NIM) (DMEM/F-12 (1:1)) (ThermoFisher Scientific), supplemented with 1x N2, 1x NEAA, and 5mM Nicotinamide. At D4, the medium was fully replaced with NIM ^66^. Beginning at D10, cultures were transitioned to retinal induction medium (RIM) consisting of DMEM/F-12 supplemented with 1x B27 without Vitamin A (ThermoFisher Scientific), 1% antibiotic-antimycotic solution (ThermoFisher Scientific), 1% GlutaMAX and 1x NEAA with daily media changes until D17. At D17, adherent cells were mechanically lifted and transferred as small clusters (<5 mm^2^) into 90mm suspension culture dishes (SUMITOMO BAKELITE CO, Ltd). Floating aggregates were subsequently cultured in RIM supplemented with IGF1 (ThermoFisher Scientific) until D41. From D42 to D62, the medium was further supplemented with 10% fetal bovine serum, 20ng/ml IGF-1, and 1 mM taurine (Sigma). Between D63 and D91, 1 μM *9-cis* retinal was added at each media change. After D91, the concentration of 9-cis retinal was adjusted to 0.5 μM and maintained until the completion of differentiation. Following the transition to suspension culture, a partial media change was carried out every 2–3 days. At each step, media were freshly supplemented with IGF-1, taurine, and 9-*cis* retinal under dim light.

### Immunohistochemistry

Human retinal organoids were fixed in 4% PFA solution (FD Neurotechnologies) containing 1% BSA for 1 hour at room temperature (RT), followed by a single wash. Samples were cryoprotected sequentially in 15% sucrose for at least 2 hours at RT and then in 30% sucrose overnight at 4 °C. Organoids were subsequently embedded in Tissue-Tek O.C.T. compound (SAKURA) and sectioned at a thickness of 10 μm. Sections were allowed to equilibrate at RT for at least 1 hour prior to staining or storage at −80 °C. For immunostaining, sections were blocked with 5% donkey serum in PBS for 1 hour at RT and then incubated with primary antibodies diluted in blocking solution overnight at 4 °C. After three 10 min washes in PBS, sections were incubated with species-specific secondary antibodies conjugated with Alexa Fluor 488 diluted in blocking solution (1:500; ThermoFisher Scientific), along with 4,6-diamidino-2-phenylindole (DAPI) for 1 hour at room temperature. Following three additional PBS washes, sections were mounted for imaging.

For rodent retinal tissues, *rd10* mice (at P36) and P23H rats (at P84) were euthanized using CO2. Eyes were enucleated, and eyecups were fixed in 4% PFA for 15 min. After removal of the cornea and lens, eyecups were further fixed for an additional 15 min at RT, followed by cryoprotection in 20% and 30% sucrose solutions (1 hour and overnight at 4 °C, respectively). Samples were embedded in O.C.T., frozen, and sectioned at 12 μm thickness. Following two PBS washes, sections were blocked for 1 hour at RT in PBST (PBS containing 5% donkey serum and 0.3% Triton X-100). The sections were then incubated with primary antibodies overnight at 4 °C. After subsequent PBS washes, the sections were incubated with appropriate secondary antibodies and 1 μg/ml of DAPI for 1 hour at room temperature. After final washes, sections were mounted for imaging.

### Image acquisition and analysis

Fluorescence imaging was performed using Nikon A1R and Leica SP8 resonant scanning confocal microscopes. Image processing and analysis were carried out using FIJI and Photoshop CC 2025 software. For quantification of rhodopsin fluorescence in retinal organoid sections, z-stack images were first converted into maximum intensity projections. Multi-channel RGB (red, green, blue) images were decomposed into individual 8-bit grayscale components. Regions of interest were defined using the “Moments” thresholding algorithm in FIJI, which was applied consistently across all samples to ensure uniform and unbiased analysis. Finally, all quantification was performed while blinded to the experimental groups.

### RNA extraction and library preparation

Total RNA was isolated from homogenized retinal organoids and retinal tissues from P23H and *rd10* animals using RNeasy Mini Kit (QIAGEN), following the manufacturer’s instructions. RNA quality was evaluated using the TapeStation system (Agilent), and only samples with high quality (RNA integrity number (RIN) ≥9) were used for the downstream applications. For library preparation, 100 ng of total RNA was used to generate strand-specific mRNA sequencing libraries using the TruSeq stranded RNA Kit (Illumina).

### RNA-seq data analysis

Illumina bulk RNAseq reads were quality checked and aligned to Ensembl reference transcriptomes of *Homo sapiens* (version 115), *Rattus norvegicus* (version 115), and *Mus musculus* (version 115) for control and treated samples of patient derived retinal organoids (*CEP290*-LCA), Rho^P23H/−^ and Pde6b^*rd10*/*rd10*^, respectively. Analysis pipeline has been described previously ^37^. Briefly, transcriptome alignments were performed with Kallisto and processed with the R package tximport, before differential expression analysis with edgeR and limma. Additional R packages like STRINGdb (for gene interaction network) and fgsea (for gene set enrichment analysis) were used for various downstream analyses. Unless specified otherwise, base R and tidyverse packages were employed for statistical analysis and visualizations.

Differentially expressed genes (DEG) were identified by setting a p-value filter of 0.05 and selecting the top 500 over and under expressing genes with at least 1.2-fold change. DEGs were mapped to KEGG pathways using gProfiler and filtered to identify pathways that had minimum 3 DEG mappings and at least 5% of the pathway impacted by differential genes.

## Supplementary Material

This is a list of supplementary files associated with this preprint. Click to download.

• MinjiKimSupplementary04232026.pdf

## Data Availability

High throughput sequencing datasets generated in this study are available through GEO accession # GSE327435. The data will be released immediately upon acceptance. For reviewers: https://www.ncbi.nlm.nih.gov/geo/query/acc.cgi?acc=GSE327435 Enter token abszokgejloftmx into the box
